# Nutrients and probiotics: current trends in their use to eradicate *Helicobacter pylori*

**DOI:** 10.3164/jcbn.20-51

**Published:** 2020-06-05

**Authors:** Osamu Handa, Yuji Naito, Motoyasu Osawa, Takahisa Murao, Hiroshi Matsumoto, Eiji Umegaki, Akiko Shiotani

**Affiliations:** 1 Department of Internal Medicine, Division of Gastroenterology, Kawasaki Medical School, 577 Matsushima, Kurashiki, Okayama 701-0192, Japan; 2 Department of Molecular Gastroenterology and Hepatology, Kyoto Prefectural University of Medicine, 465 Kajii-cho, Kamigyou-ku, Kyoto 602-8566, Japan

**Keywords:** *Helicobacter pylori*, food factors, probiotics, cancer chemoprevention

## Abstract

*Helicobacter pylori* is a well-known bacterium that infects the human gastric mucosa and causes gastric inflammation, ultimately resulting in gastric cancer. To reduce the incidence of gastric cancer, eradication therapy is important. However, the rate of successful eradication gradually decreases due to increased antibiotic resistance to *Helicobacter pylori*. In order to increase the eradication rate and reduce gastric cancer incidence, food factors or probiotics are expected to play a beneficial role. Although several foods have been reported to inhibit bacterial load and gastric inflammation, further assessment on large population prospective studies in this field is warranted. Several food compounds, including phytochemicals, are reported to suppress the incidence of gastric cancer. Future evaluations should consider differences in geographic factors. Probiotics are effective and safe for use in *Helicobacter pylori* eradication therapy.

## Introduction

*Helicobacter pylori* (*H. pylori*) is a bacterium that can infect the human stomach. Usually, *H. pylori* is not eliminated by our immune systems, causing continuous inflammation within the stomach, and resulting in atrophic changes to the mucosa. It is well documented that *H. pylori* plays a role in the development of gastric cancer.^(1–3)^ Eradication therapy for *H. pylori* can significantly reduce the gastric cancer incidence as assessed by meta-analysis.^(4)^ Therefore, eliminating *H. pylori* infection by eradication therapy is important in order to reduce the prevalence of developing gastric cancer. Eradication therapy involves the combined use of two or more drugs including antibiotics, however, the successful eradication rate is reported at around 70–85%; which is not high enough to fulfill the criteria of successful treatments of this curable infectious disease.^(5)^ Moreover, the effect of eradication therapy can sometimes result in patients experiencing side effects such as diarrhea, allergic eruption, and nausea. In a few cases, critical hemorrhagic enterocolitis^(6)^ or anaphylaxis reaction^(7)^ is experienced. Several food factors or probiotics are suggested to increase the eradication rate of *H. pylori* infection and reduce the incidence of side effects. In this review, we summarize the current issues of* H. pylori* eradication therapy and the beneficial effects of food factors and probiotics to help increasing eradication rate.

## Current Issues in *H. pylori* Eradication Therapy

In Japan, the medical management strategies for the primary eradication of *H. pylori* involves a 7-day treatment with a combination of three drugs; vonoprazan (VPZ) or an available proton pump inhibitor (PPI), clarithromycin (CAM), and amoxicillin (AMPC). However, the *H. pylori* eradication rates under this form of combination therapy have been gradually declining each year, largely attributed to the rise in *H. pylori* drug resistance; particularly to CAM, a drug widely used in primary eradication therapy.^(8)^ The eradication rate with the combined drug treatment PAC; consisting of PPI, AMPC and CAM, is approximately 70%. However, following the 2015 Japanese healthcare approval of the combined drug treatment VAC; consisting of VPZ, AMPC and CAM, this therapy significantly improved the *H. pylori* eradication rate to approximately 90%. Although the apparent incidence of side effects caused by VAC does not differ from PAC,^(9)^ VAC is reported to have a greater impact on influencing the intestinal microbiota compared to PAC.^(10)^ Therefore, an *H. pylori*-selective eradication drug treatment strategy would be ideal to minimize the potential detrimental effects of modulating the gut microbiota.

## Antimicrobial Properties of Food Factors

Several reports have suggested the antimicrobial activity of food factors on *H. pylori*. Recently β-caryophyllene, a volatile bicyclic sesquiterpene compound that can be present in the essential oils of many edible plants such as cloves, oregano, and cinnamon, has been reported to significantly inhibit *H. pylori* growth via the downregulation of virulence factors in a model using Mongolian gerbils. It also inhibits cytotoxin-associated gene product A (CagA) protein translocation from *H. pylori* to AGS gastric cancer cell line via the inhibition of type IV secretion system expression,^(11)^ a needle like apparatus of *H. pylori*.^(12)^

A Chinese study comparing dietary patterns on *H. pylori* infection rates revealed that a “grains-vegetables” dietary pattern is associated with a decreased risk of infection [odds ratio (OR) = 0.82; 95% confidence interval (CI): 0.732–0.973; *p* = 0.04], whereas a “high-salt” dietary pattern is associated with an increased risk of *H. pylori* infection (OR = 1.13; 95% CI: 1.004–1.139; *p* = 0.048).^(13)^ Another study demonstrated that a diet rich in carbohydrates and with a high sugar content was positively associated with the prevalence of *H. pylori* infection (OR = 1.65; 95% CI: 1.27–2.17; *p*<0.001).^(14)^ In contrast, a diet characterized by a high intake of animal offal, animal blood, fish, seafood, and poultry was associated with a reduction in the prevalence of *H. pylori* infection (OR = 0.75; 95% CI: 0.57–0.98; *p* = 0.04).^(14)^ These findings suggest the possible influence dietary patterns and different types of foods can have on *H. pylori* itself. However, these results were not corroborated on a Lebanese population (*n* = 294) where no relationship between dietary habits and *H. pylori* infection were observed.^(15)^

A number of food factors could possess antimicrobial effects against *H. pylori*. Future efforts to establish the potential benefits of these natural food compounds are warranted in order to develop more effective eradication therapies.

## Food Factors and Gastric Cancer Incidence

**Fig. 1:**
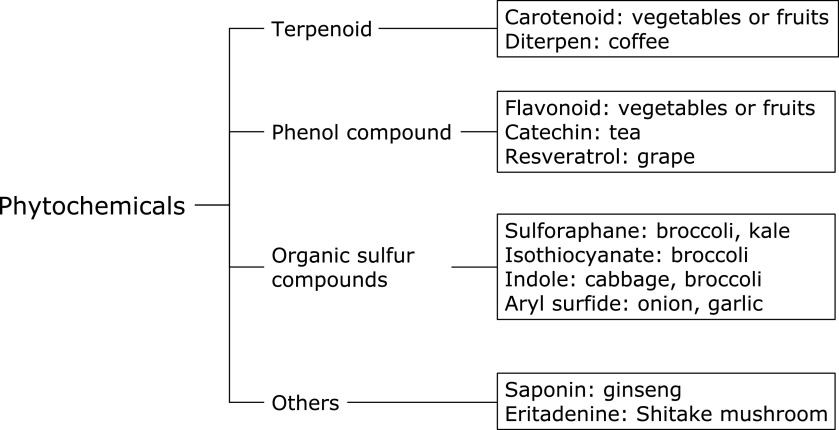
Phytochemicals.

Determining whether consumption of food products or supplements may be beneficial for preventing the incidence of developing gastric cancer incidence is of great interest. Evidence to date strongly supports the notion that diet can play a critical role in defining the outcome of *H. pylori* infection, particularly if certain dietary components are taken on a regular basis for a long period of time. Amongst the various constituents present in natural foods, phytochemicals have been widely investigated for their beneficial effects. Figure 1 illustrates the typical phytochemical classification.

According to several epidemiological studies, dietary carotenoids (lycopene astaxanthin or β-carotene) have been shown to have preventive effects on gastric inflammation due to their antioxidant properties. Moreover, antioxidant and anti-inflammatory effects of carotenoids may play a role in decreasing *H. pylori*-induced gastric inflammation. As for lycopene, a subclass of dietary carotenoids abundant in fruits and vegetables, a significant inverse association between total dietary lycopene and gastric cancer risk was observed (OR = 0.60; 95% CI 0.42–0.85; *p* = 0.012)^(16)^ in a Korean case control study. This significant association between dietary lycopene intake and gastric cancer risk was also observed in *H. pylori*-positive subjects. As the cancer suppressive effect of lycopene has been reported in various cancers, such as lung, colon, or prostate cancer, this suppression is not influenced by the presence of *H. pylori*. Astaxanthin possesses strong anti-oxidation properties.^(17,18)^ The *H. pylori*-induced upregulation of oxidative stress in gastric epithelial cell was inhibited following supplementation with astaxanthin.^(17)^ Additionally in an *H. pylori*-infected mice model, astaxanthin reduced bacterial load and gastric inflammation.^(18)^ β-Carotene is reported to suppress reactive oxygen species-mediated inflammatory signaling; including mitogen-activated protein kinases and redox-sensitive transcription factors, in infected tissues as well as reducing expression of inflammatory mediators, interleukin-8, inducible nitric oxide synthase, and cyclooxygenase-2.^(19)^

Several case-control experimental and epidemiological studies have suggested that consumption of garlic may reduce gastric cancer risk and suppress *H. pylori* growth. A blinded randomized placebo controlled trial in China showed that *H. pylori* eradication using a two week combined treatment with AMPC and PPI, followed by vitamin (C, E, and selenium) or garlic (extract and oil) supplementation for seven years, was associated with a statistically significant reduction in the mortality risk of gastric cancer for more than 22 years (OR = 0.64; 95% CI 0.46–0.91 or OR = 0.57; 95% CI 0.57–1.13 respectively).^(20)^ However, a recent paper evaluating two large prospective cohort studies in US on 77,086 women and 46,398 men respectively, report that a high garlic intake did not reduce risk of gastric cancer,^(21)^ suggesting the importance of future work in this field of research to be carried out as large population prospective studies. Furthermore, attention should be paid to geographical differences influencing the gastric cancer risk.

## Probiotics and *H. pylori*

The direct or indirect interaction of gastrointestinal bacteria with *H. pylori* has been explored. The human gastric isolate *Lactobacillus rhamnosus* UCO-25A (*L. rhamnosus* UCO-25A) formed biofilms on abiotic and cell surfaces to modulate the inflammatory response triggered by *H. pylori* on AGS human gastric cancer cell line and THP1 human monocytic leukemia cell line *in vitro*. In addition, *L. rhamnosus* UCO-25A-formed biofilm significantly inhibited *H. pylori* infection in AGS cells.^(22)^ Another group reports the effect of honey-derived *L. rhamnosus* to provide benefits similar to clarithromycin, in terms of reducing *H. pylori* infection and gastritis in C57BL/6 mice model.^(23)^

*Lactobacillus reuteri* (L. reuteri) is reported to increase the *H. pylori* eradication rate irrespective of CYP2C19 genotype and antibiotic resistance pattern in a randomized placebo-controlled study.^(24)^ In this study, *L. reuteri* exhibited significant improvement in the eradication rate after 14-day high PPI-bismuth-containing quadruple therapy. However, this was not observed after 7-day therapy, thereby questioning the variability in the effect of *L. reuteri*.

We also reported that *Clostridium butyricum* MIYAIRI 588 significantly improve the eradication rate and the side effect is not different.^(25)^

Meta-analysis data has shown that in the comparison of probiotics vs placebo,^(26)^ probiotics monotherapy had higher eradication rate than placebo (OR = 7.91; 95% CI: 2.9–21.0, *p*<0.001), although the mean weighted eradication rate was 14% (95% CI: 2–25%, *p* = 0.02). Since no significant difference in adverse events was found in this analysis between probiotics and placebo (OR = 1.0; 95% CI: 0.06–18.08), this prompts the suggestion that monotherapy of *H. pylori* compared to placebo is safe but minimally effective.

The effect of probiotics on the eradication of *H. pylori* has been explored in a meta-analysis study, that demonstrated the rates of eradication and adverse events were 84.1 and 14.4% in probiotic additional group, while 70.5 and 30.1% in were observed in the eradication only group.^(27)^ Collectively, these studies suggest that probiotics are useful to improve *H. pylori* eradication therapy.

## Conclusion

Although many studies evaluating the role of dietary ingredients on *H. pylori* or gastric cancer have been performed, further studies involving large-scale clinical trials are needed to obtain a better understanding of the precise role played by dietary ingredients in *H. pylori*-associated pathogenesis.

## Author Contributions

OH and YN contributed to the original concept of the manuscript. MO, TM, HM collected papers. OH wrote the initial draft of the manuscript. EU and AS revised the manuscript critically for important intellectual content.
